# Multimodal NASH prognosis using 3D imaging flow cytometry and artificial intelligence to characterize liver cells

**DOI:** 10.1038/s41598-022-15364-7

**Published:** 2022-07-01

**Authors:** Ramkumar Subramanian, Rui Tang, Zunming Zhang, Vaidehi Joshi, Jeffrey N. Miner, Yu-Hwa Lo

**Affiliations:** 1 Department of Electrical and Computer Engineering, University of California San Diego, La Jolla, CA 92093 USA; 2 Viscient Biosciences Inc, San Diego, CA 92121 USA

**Keywords:** Computational models, Cellular imaging

## Abstract

To improve the understanding of the complex biological process underlying the development of non-alcoholic steatohepatitis (NASH), 3D imaging flow cytometry (3D-IFC) with transmission and side-scattered images were used to characterize hepatic stellate cell (HSC) and liver endothelial cell (LEC) morphology at single-cell resolution. In this study, HSC and LEC were obtained from biopsy-proven NASH subjects with early-stage NASH (F2-F3) and healthy controls. Here, we applied single-cell imaging and 3D digital reconstructions of healthy and diseased cells to analyze a spatially resolved set of morphometric cellular and texture parameters that showed regression with disease progression. By developing a customized autoencoder convolutional neural network (CNN) based on label-free cell transmission and side scattering images obtained from a 3D imaging flow cytometer, we demonstrated key regulated cell types involved in the development of NASH and cell classification performance superior to conventional machine learning methods.

## Introduction

Non-alcoholic steatohepatitis (NASH) is a major contributor to morbidity and mortality. Ongoing injury, altered wound healing, and uncontrolled extracellular matrix (ECM) accumulation can lead to liver fibrosis and the extent of fibrosis can progress to liver cirrhosis and liver failure^1,2^. Histopathological analysis of liver biopsies remains the gold standard for the diagnosis of fibrosis but this approach is hindered by inter/intra-observer variability and overlooks 3D (three-dimensional) structural changes resulting from functional alternations^3–5^. Thus, there is an unmet clinical need to understand the molecular & structural level changes in LEC and HSC cells in NASH subjects and identify single-cell imaging & texture-related biomarkers to complement traditional histological classification^6,7^.

In the last two decades, several novel diagnostic methods and prognostic biomarkers have been studied by different research groups to help identify NASH. The first line of diagnosis is using liver enzymes as a biomarker to identify the ratio of alanine transaminase (ALT) to aspartate aminotransferase (AST) and elevated fragments of plasma cytokeratin (CK18)^8–10^. But the sensitivity and specificity are not sufficient because the ALT/AST ratio is also elevated during other diseases such as viral hepatitis. An alternate leading quantitative approach is to identify the presence of fat in liver tissue by measuring liver stiffness as a surrogate biomarker. Several non-invasive diagnostic methods such as ultrasound, magnetic resonance imaging (MRI), transient elastography, ultrasound elastography, magnetic resonance elastography (MRE), MR apparent diffusion coefficient (ADC), magnetic resonance imaging (MRI) with proton density fat fraction (PDFF) and proton magnetic resonance spectroscopy^11–13^ have been recently developed in the last decade as a non-invasive diagnostic tool to access the percentage of fat in the liver. However, these methods are not scalable due to high cost, and more importantly, the fat content alone does not indicate liver inflammation and tissue fibrosis^14^. Recently, there has been an increasing interest in digital pathology & imaging techniques^3,15,16^. Over the past decade, imaging the entire tissue section has made a significant stride with the advance of digital pathology and in-depth characterization of thousands of molecules using matrix-assisted laser desorption/ionization (MALDI) mass spectrometry^17^. To enhance the sensitivity of the above-mentioned biomarkers, deep learning-based methods have recently been applied to imaging data, clinical and omics (genetics, transcriptomic, proteomic, and metabolomic) data, and patients' electronic health records (EHR)^18–23^. While these methods study different biomarkers, they do not unravel quantitative image features and their relationship to histopathologic features, which link them to NASH disease outcomes. In our study, we propose a multimodal imaging approach that can provide temporal and spatial changes of LEC and HSC and combine these images with artificial intelligence to characterize liver cells at single-cell resolution.

NASH is a complicated disease with multiple pathways and an emerging cause of liver-related mortality with no reliable biomarkers to predict disease progression. We propose a platform that utilizes a 3D imaging flow cytometer (3D-IFC) with fluorescent channels, transmission, and side scattered images at single-cell resolution with a customized autoencoder CNN based on dual-modality image input. We studied key cell types involved in NASH using single-cell imaging and 3D digital reconstructions of HSC and LEC cells, with the aim of establishing a multi-modality molecular liver imaging platform. The aim of this platform is to advance liver cell analysis and build better predictive clinical models, incorporating multiple textures and intensity-based features that correlate highly with disease progression. We also compared the performance of conventional machine learning models with the performance of our customized autoencoder CNN model and demonstrated that our customized CNN model substantially outperforms the conventional methods in HSC and LEC cell classification.

## Methods

### Cell sample preparation

Cell samples were harvested and prepared from two healthy donors and two NASH patients. The cell samples were provided by Viscient Biosciences Inc. Viscient Biosciences Inc. provides assurances that the cells come from tissues collected in compliance with applicable laws and provided based on informed consent by the donors. Frozen vials of HSC, LEC cells, and hepatocytes were thawed in a 37 degree Celsius water bath. HSC and LEC cells were then transferred to 5 ml of their respective media—HSC in DMEM+10% FBS, LEC cells in EGM2 media from Lonza (Catalog# CC-3162). HSC and LEC cells were then spun at 200g for 5 minutes. The supernatant was aspirated, and the pellet was resuspended in 1ml of media. Hepatocytes were thawed in Gibco hepatocyte thaw media (Catalog number: CM7500) per the manufacturer's instructions. They were then transferred to 2 ml of Williams E maintenance medium with no phenol red (Catalog number: A1217601, Thermo Fisher). Gibco Williams E basal media was supplemented with primary hepatocyte maintenance supplements (Catalog number# CM4000) to make complete maintenance media used for tissue maintenance.

### 3D-IFC dual-modality imaging system

**Figure 1:**
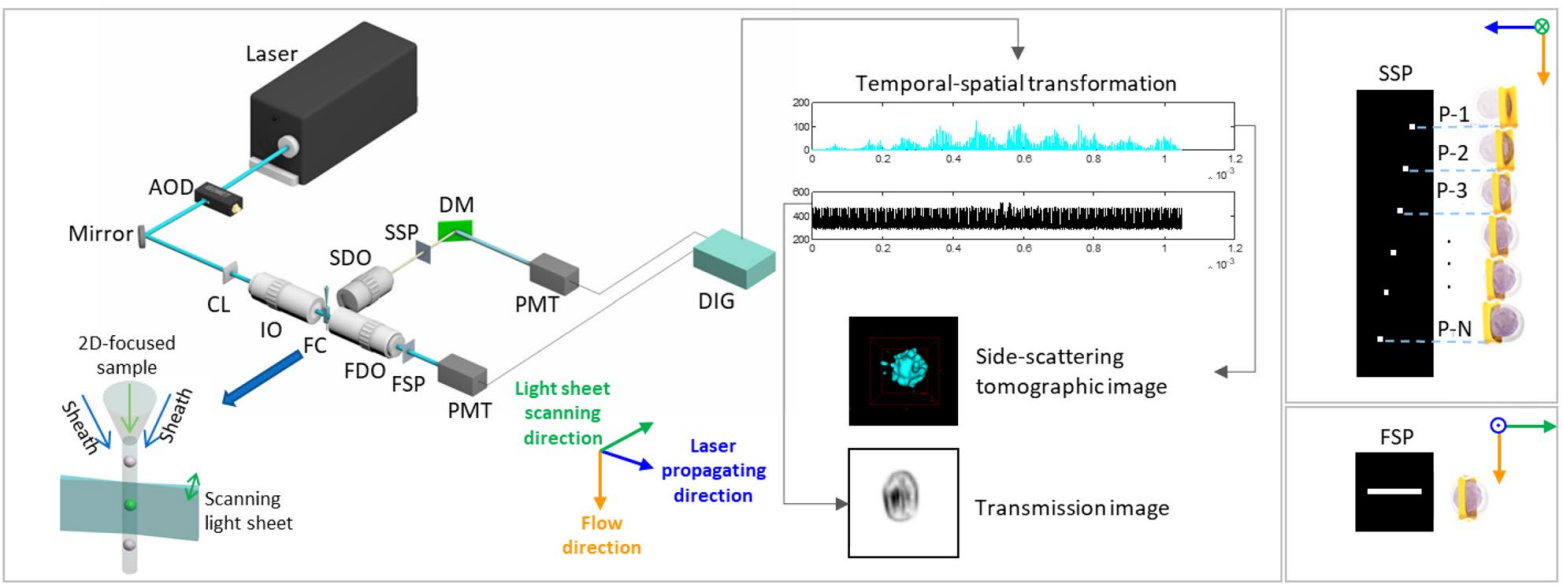
3D imaging flow cytometer (3D-IFC) dual-modality imaging system. AOD, acousto-optic deflector; CL, cylindrical lens; IO, 20X/0.42NA illumination objective; SDO, 10X/0.28 NA side detection objective; SSP, side spatial filter; DM, dichroic mirror; FC, flow cell; FDO, 50X/0.55 NA forward detection objective; FSP, forward spatial filter; PMT, photomultiplier tube; DIG, 25MSps digitizer.

The 3D-IFC dual-modality imaging system design is illustrated in Fig. 1. A 2D hydrodynamically focused single-cell stream was interrogated by a fast-scanning light sheet illumination at a scanning rate of 200 kHz. Two spatial filters with different designs were placed on the image planes of side and forward detection objectives, respectively. The side spatial filter (SSP) contains a series of spatially positioned pinholes aligned with the vertical cell flow streams with a preset horizontal separation. When the cell passes through the SSP, the three-dimensional volume of the cell is optically sectioned chronologically by the combination of the scanning light sheet, cell flow, and the pinhole series^24^. In the meantime, the light transmitted through the cell is also spatially filtered by the forward spatial filter (FSP). As the cell stream flows through the optical interrogation area, temporal PMT signals are generated from side-scattered and transmitted light and encoded by the SSP and FSP, respectively. The encoded side scattered signals generate 3D side-scattering tomographic images and the encoded transmitted signals generate 2D transmission images of cells by mapping the timing and magnitude of temporal readouts to positions and intensities of voxels/pixels using the temporal-spatial transformation^25^.

### 3D-IFC image acquisition experiment

**Figure 2:**
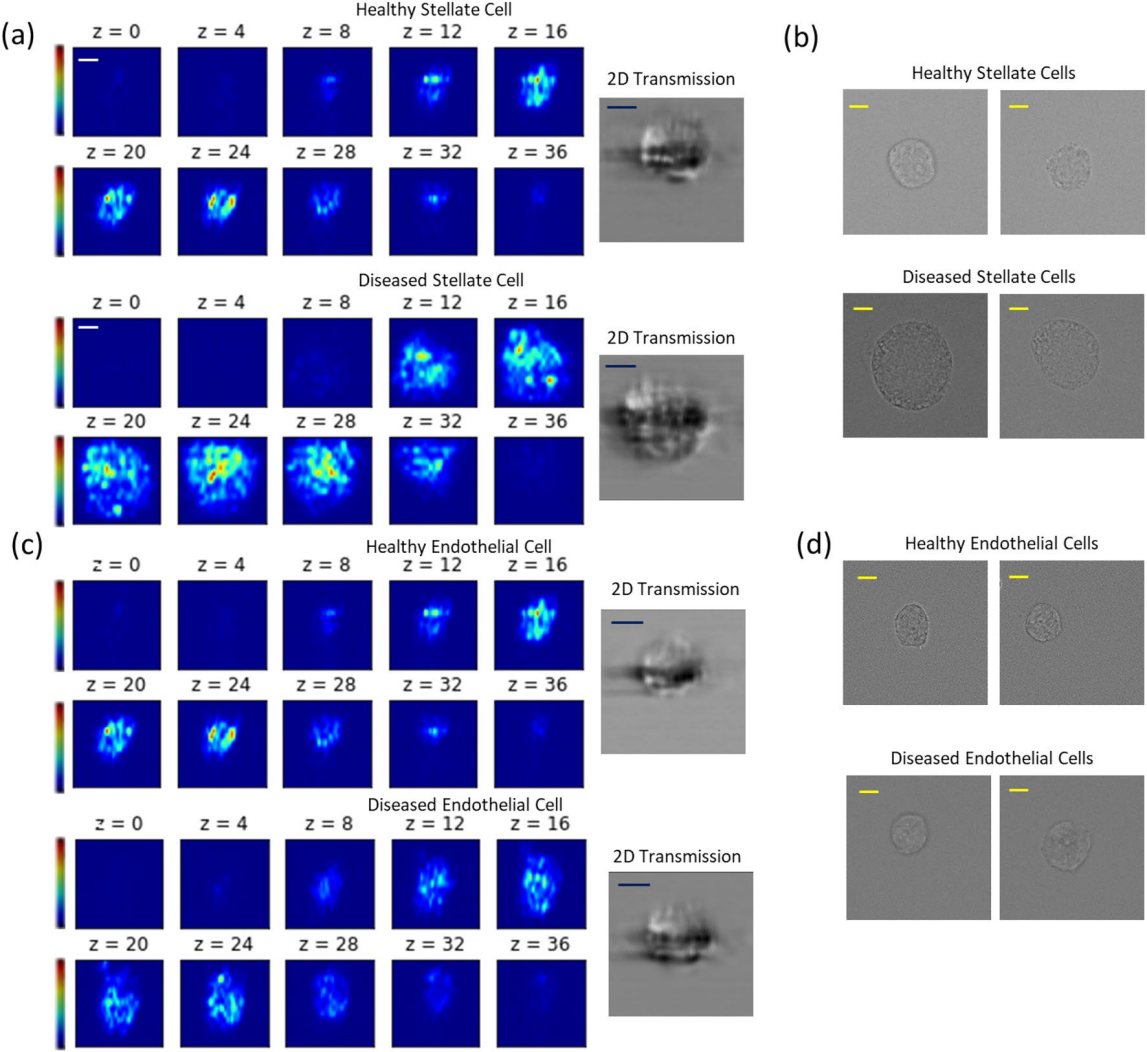
2D transmission and 3D SSC images of liver cells by 3D-IFC. (**a**) Example 3D SSC image stacks of healthy and diseased HSC cells and corresponding 2D transmission images. (**b**) Example microscope images of healthy and diseased HSC cells. (**c**) Example 3D SSC image stacks of healthy and diseased LEC cells and corresponding 2D transmission images. (**d**) Example microscope images of healthy and diseased LEC cells. Scale bar: 10 µm.

For the image acquisition experiment, each batch of cell samples was independently run by the 3D-IFC system. HSC and LEC cells samples were analyzed separately for every subject. Each cell produced an image pair of a 2D transmission and a 3D side-scattering image. The captured 3D side-scattering image was stored as a 3D image stack of 40 × 40 × 40 voxels with a field of view of 40 × 40 × 40 µm^3^. The acquired 2D transmission was stored to 80 × 80 pixels with a field of view of 40 × 40 µm^2^. The representative LEC and HSC cells acquired from the 3D-IFC system are presented in Fig. 2.

### Data preparation and image processing

In the data preparation process, we first removed the background noise for both transmission and side-scattering images, leaving only the pixels of interest through the intensity thresholding. Then, the pixel intensities of the transmission and side-scattering images were normalized globally to the highest intensity level of the dataset. To address the class imbalance issues, we randomly sampled 5000 image pairs from the overall population for each cell type and each subject to balance the class occurrence frequencies^26^. The total image dataset size for a cell type is 20,000 image pairs.

### Texture and geometrical morphological characterization

After randomly sampling 5000 image pairs for each cell type and each subject, the algorithm extracts several morphometric and textural features. Cell shape-related geometrical features, including cell area, perimeter, cell volume, surface area, etc., were extracted from the 2D transmission and 3D side-scattering images using the image processing algorithms. Haralick texture features were calculated from a Gray Level Co-occurrence Matrix (GLCM)^27,28^, a matrix that counts the co-occurrence of neighboring gray levels in the image. It consists of two steps for feature extraction. The GLCM was computed in the first step, while the texture features based on the GLCM were calculated in the second step. GLCM characterizes the texture of an image in grey scale by calculating how often pairs of pixel with specific values and in a specified spatial relationship occur in an image. It is one of the most popular second-order statistical features. Then, second-order statistics estimate the properties of two or more pixel values occurring at specific locations relative to each other. For 2D images, typical values used for “*d*” equal{1, 2, 3, 4} and those for “θ” equal {0° , 45° , 90° , 135° }. On the other hand, for the 3D operation, the total number of GLCMs is 26 (thirteen directions and four offsets). Moreover, we calculated the average texture feature to evaluate the value for each cell type. *Z*-score normalization was employed on each of the feature vectors, which converted the features to zero mean and unit variance. The mean and standard deviation (σ) of the feature vector is calculated as follows:

1 $${r}_{n}= \frac{r - mean}{\sigma }$$

where *r* is the original value, ${r}_{n}$ is the new value, and the mean and σ are the mean and standard deviation of the original data, respectively. Feature extraction algorithms are built using the comprehensive set of reference-standard algorithms provided by the Image Processing Toolbox in MATLAB 9.11 (R2021b).

The complete list of morphological features is included in the supplementary materials. In total, 55 morphological features were extracted, including 28 features from the 2D transmission image and 27 features from the 3D side-scattering image.

### Conventional Machine learning for cell characterization

The morphological features extracted from cell images were used to explore the possibility of characterizing the disease status of the cell. Traditional machine learning algorithms were used to establish a benchmark for cell status classification. We applied seven conventional machine learning classifier models to test the classifier performance. The stratified 4-fold cross-validation (CV) approach was used to pre-process the image features dataset to train the classifier models and evaluate their performance. For each subject, we conducted an 80/20 train/test split for the model training and validation process. The model was then validated using the instance from the validation set. All the models adopt the same data pre-processing method. The morphological features were used as the model input for the classifier models. The classifier models output the disease status of the cell (healthy/diseased).

### Customized UNet CNN for cell characterization

**Figure 3:**
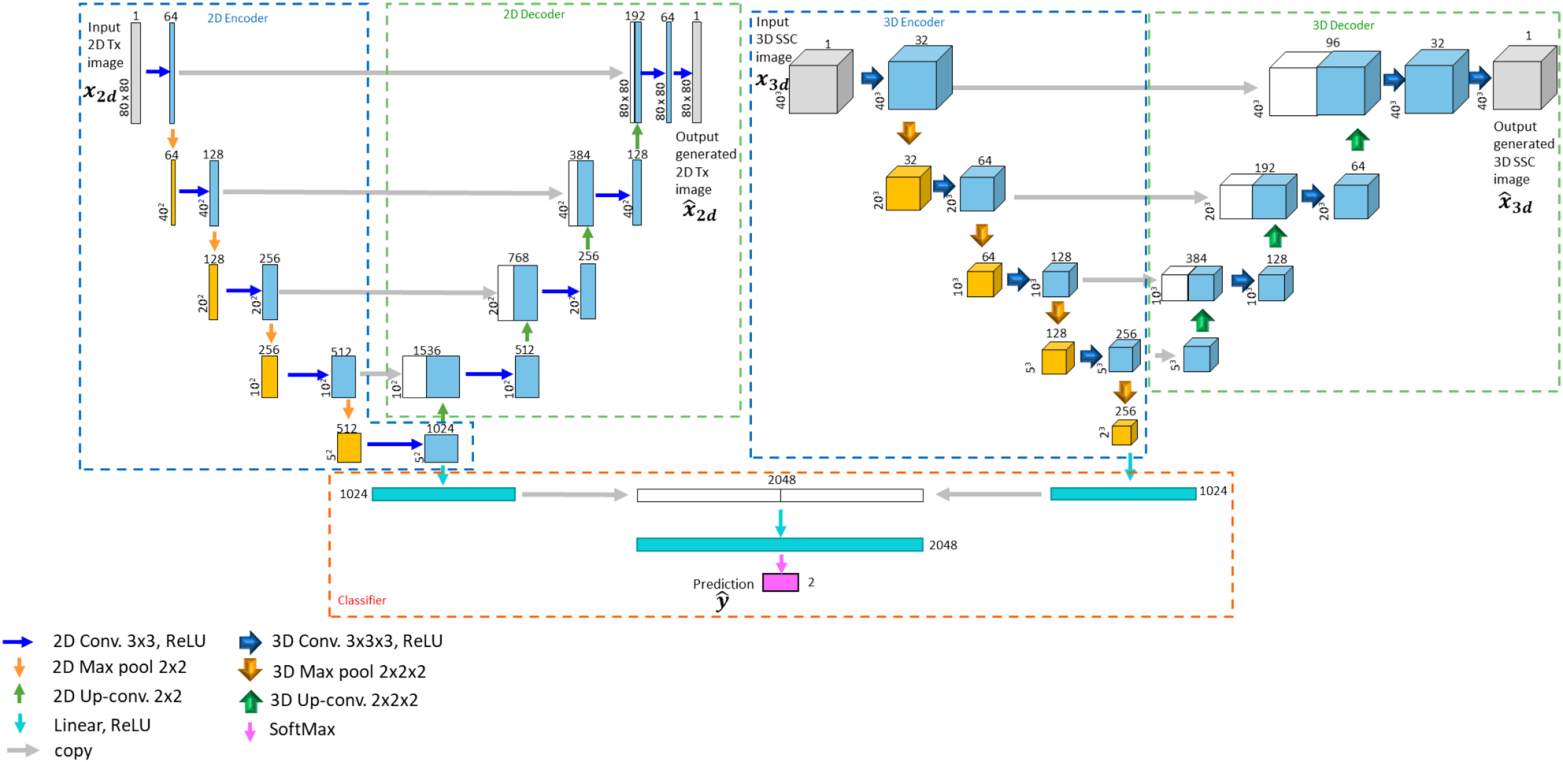
Fused UNet autoencoder model architecture.

Although conventional feature extraction methods generate human interpretable cell features, the features we extracted might not be the most representative of differentiating groups, and it is possible to eliminate the most distinguishable features among groups through the subjective feature extraction pipeline. To exploit the high information content in the dual-modality cell images from the 3D-IFC system, a customized autoencoder model, Fused UNet, was developed based on UNet architectures introduced by our previous work^29^. Our previous work examined the utilization of side-scattering images for cell classification. However, the side-scattering image only reflects the 90-degree scattering light profile of the cell, while the light loss information caused by the occlusion of the cell when it passes through the optical interrogation area can only be captured by the 2D transmission image. Therefore, combining the two imaging modalities could provide a complete light profile information of the cell, which leads to our 'Fused UNet' architecture. Fused UNet architecture combines two UNet structures (2D UNet and 3D UNet), each taking a single image modality as the input. Image features are extracted by the convolutional layers and encoded to subsequent layers by the max-pooling kernels during the contracting paths. The latent spaces from the two UNets are concatenated together with a Softmax layer to make a classification decision for the classifier. The images generated by the upsampling paths are also used to optimize the loss function during the model training process. The Fused UNet structure is illustrated in Fig. 3. The output of the Softmax layer can be written as

2 $${\widehat{{\varvec{y}}}}_{{\varvec{i}}}=\frac{{e}^{{{\varvec{x}}}_{{\varvec{i}}}}}{{\sum }_{j=1}^{C}{e}^{{{\varvec{x}}}_{j}}},i=\mathrm{1,2},\dots ,C$$

where ${\varvec{x}}$ is the input image pair vectors, $C$ is the number of classes.

The Fused UNet uses a weighted loss that consists of the mini-batch averaged cross-entropy loss (Eq. (3)) between the ground truth class and the predicted class and the weighted mean-square error loss (Eq. (4)) between the input and generated output images pixel values. The averaged cross-entropy loss ${L}_{CE}$ can be expressed as

3 $${L}_{CE}=-\frac{1}{N}\sum_{i=1}^{N}{{\varvec{y}}}_{{\varvec{i}}}({\varvec{x}})\cdot \mathrm{log}({\widehat{{\varvec{y}}}}_{{\varvec{i}}}({\varvec{x}}))$$

where ${{\varvec{y}}}_{{\varvec{i}}}$ is the ground truth class vector, ${\widehat{{\varvec{y}}}}_{{\varvec{i}}}$ is the predicted class vector, and $N$ is the data size in the mini-batch.

The mini-batch averaged mean-square error loss ${L}_{MSE}$ can be expressed as

4 $${L}_{MSE}\left({\varvec{x}},\widehat{{\varvec{x}}}\right)={w}_{1}{L}_{MSE,2d}+\left(1-{w}_{1}\right){L}_{MSE,3d} = \frac{1}{N}\sum_{j=1}^{N}\left(\frac{{w}_{1}}{{M}_{2d}}\sum\nolimits_{i=1}^{{M}_{2d}}{\left({x}_{2d,i,j}-{\widehat{x}}_{2d,i,j}\right)}^{2}+\frac{{1-w}_{1}}{{M}_{3d}}\sum\nolimits_{i=1}^{{M}_{3d}}{({x}_{3d,i,j}-{\widehat{x}}_{3d,i,j})}^{2}\right)$$

where ${\varvec{x}}$ and $\widehat{{\varvec{x}}}$ are the input and generated image pair vectors, respectively, ${M}_{2d}$ is the flattened transmission image vector dimension, ${M}_{3d}$ is the flattened side-scattering image vector dimension, ${L}_{MSE,2d}$ is the mean-square error loss between the input and generated transmission image, ${L}_{MSE,3d}$ is the mean-square error loss between the input and generated 3D side-scattering image, $N$ is the data size in the mini-batch, ${w}_{1}$ is the mean-square error loss weight assigned to the two image modalities.

The weighted total loss $L$ is defined as

5 $$L={{w}_{2}\cdot L}_{CE}+\left(1-{w}_{2}\right)\cdot {L}_{MSE}$$

where ${w}_{2}$ is the weight coefficient to balance the loss between the averaged cross-entropy loss and the mean-square error loss.

**Figure 4:**
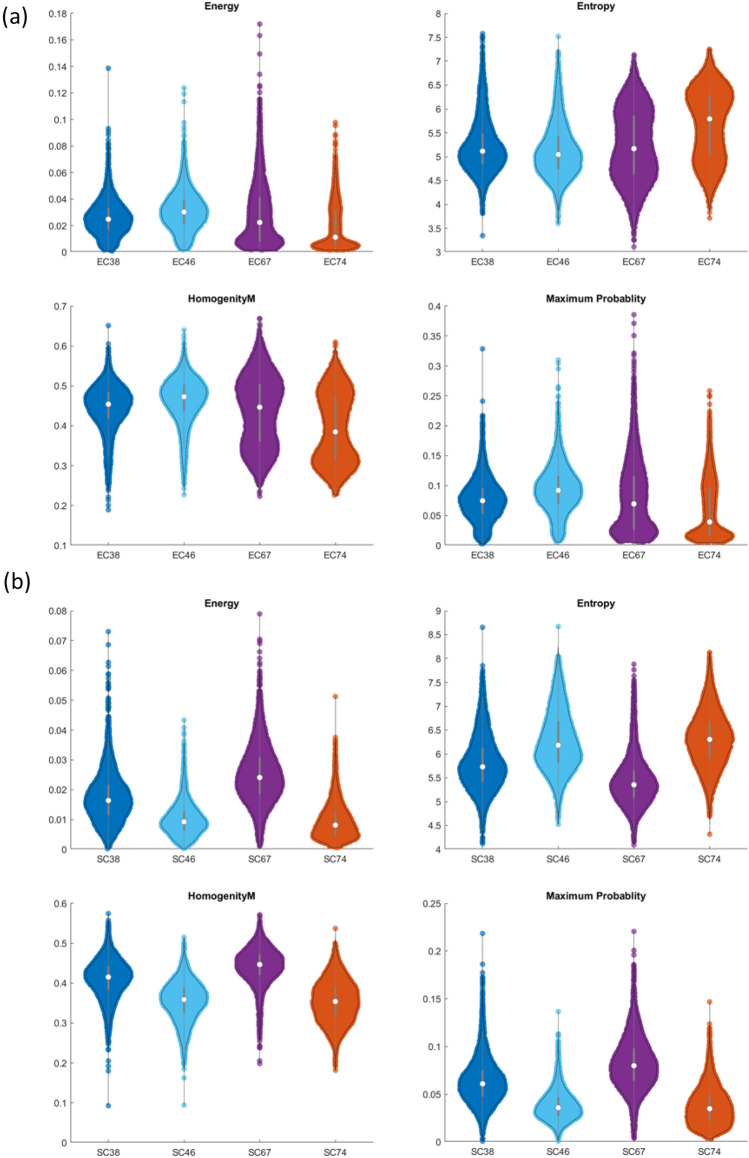
Harlick texture and geometrical features comparison of LEC and HSC healthy vs diseased cells using 2D transmission image. (**a**) Haralick texture feature comparisons such as energy, entropy, homogeneity and maximum probability of LEC healthy vs diseased cells (EC38, EC67 vs EC46, EC74) using 2D transmission image. (**b**) Haralick texture feature comparisons such as energy, entropy, homogeneity and maximum probability of HSC healthy vs diseased cells (SC38, SC67 vs SC46, SC74) using 2D transmission image.

**Figure 5:**
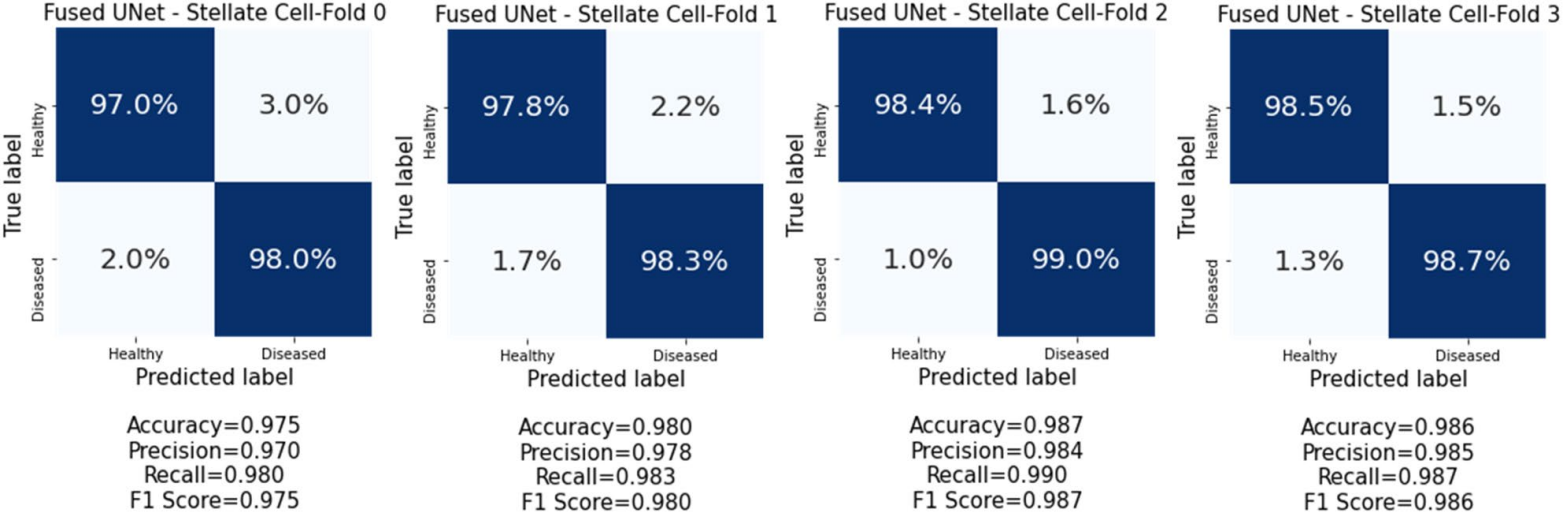
Confusion matrices from cross-validation experiments on the HSC characterization dataset.

The same stratified 4-fold cross-validation (CV) approach used in the conventional machine learning approach is also applied to prepare the image pair dataset for the training of the Fused UNet autoencoder model and the performance evaluation. The predictions made on the validation set were summarized in a confusion matrix per fold, and all confusion matrices are shown in Figs. 4 and 5. More information on the CV training curves can be found in the supplementary materials. In addition to the confusion matrix, the balanced classification precision, recall, and F_1_ score are reported for cell morphology characterization. The balanced accuracy $\overline{\sigma }$ is the arithmetic mean of class-specific accuracies and is calculated as

6 $$\overline{\sigma }=\frac{1}{C}\sum_{i=1}^{C}{\sigma }_{i}$$

where ${\sigma }_{i}$ is the class-specific accuracy, and $C$ is the number of classes.

For the cell characterization experiment, the Fused UNet model was trained for 50 epochs with the Adam optimization algorithm^30^. The initial learning was set to 1 × 10^−5^ for the first five epochs and then increased to 5 × 10^−5^ to avoid getting stuck into the local minimum. The exponential decay parameters for the Adam optimizer were set as β1 = 0.99 and β2 = 0.9999. To allow the optimization to converge, the learning rate was reduced by half if the validation metrics stopped improving for five epochs.

The Fused UNet model was implemented using the PyTorch framework and was trained on a 12-core machine with an Intel^®^ Core™ i9-10920X processor and an NVIDIA Titan RTX GPU with 24GB of VRAM.

## Results

### Histological validation of NASH

Diseased cells were isolated from donor liver tissue obtained from NASH patients. Donor's livers were scored by a trained pathologist using Batts-Ludwig methodology and NASH CRN scoring system on steatosis, inflammation, fibrosis, and NAFLD.

### Morphological characterization based on 2D transmission and 3D side scattering

**Figure 6:**
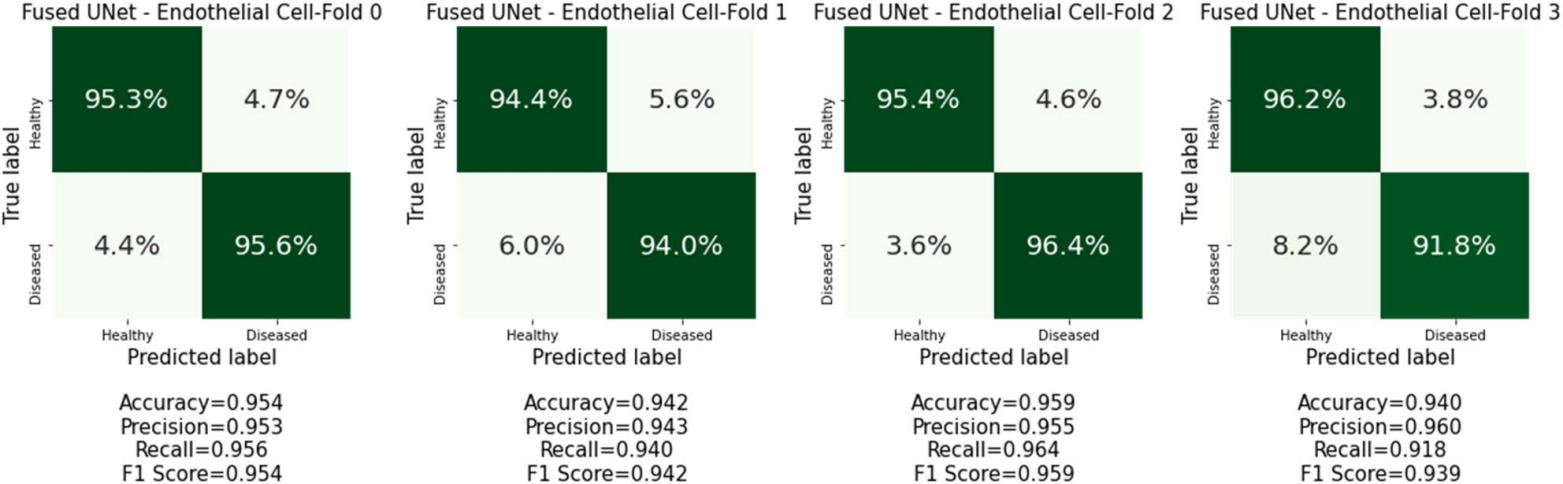
Confusion matrices from cross-validation experiments on the LEC cells characterization dataset.

After randomly sampling 5000 image pairs for each cell type and each subject, the algorithm extracts several morphometric and textural features. The LEC and HSC cells image data used in this study were collected from healthy (n = 2) and biopsy-proven NASH subjects (n = 2), both with the aim of investigating the use of image-based biomarkers as a surrogate endpoint for early diagnosis of NASH. A total of 25 features, consisting of a combination of size, shape, first-order, and second-order statistical texture measures, were computed. The morphometric features such as volume, surface area, and major/minor axis using elliptical fit were extracted to distinguish the healthy versus NASH cell types due to inflammation. We also measured texture-based features to use spatial distribution of intensity that is associated with pathological conditions such as nuclear vacuolation, a common histological characteristic in NASH. Statistical student-t test and the violin plot to show the distribution of prominent features such as energy, entropy, homogeneity, and maximum probably were plotted and presented in Fig. 6 for both LEC and HSC cells. Our analysis shows that the morphometric and texture homogeneity features for healthy and diseased LEC and HSC cells show differences in some features but similarities in others. It is unclear whether one can establish any intuitive, human recognizable features to distinguish between healthy and diseased cells for NASH reliably. This finding motivates the development of deep learning approaches discussed next.

### Customized UNet CNN cell characterization based on the dual-modality image input

We conducted the cell characterization on both HSC and LEC cells datasets using the customized UNet CNN model. The confusion matrices for HSC and LEC cells characterization are presented in Figs. 5and6, respectively. The results show that our Fused UNet model was able to characterize both HSC and LEC cells with high accuracy between NASH and healthy donors with the dual-modality image inputs acquired by the 3D IFC system. For HSC characterization, Fused UNets return a high balanced characterization F_1_ score (0.975–0.987, mean F_1_ score: 0.982) for all CV folds. The characterization of LEC cells generally returns a balanced characterization F_1_ score (0.939–0.959, mean F_1_ score: 0.9485) for all CV folds. Our result shows that we can separate the cells based on the physical structure, which suggests that there are fundamental changes that occur during the disease process that alter the structure and likely the function of these cells during disease progression.

### Conventional machine learning for cell characterization

**Figure 7:**
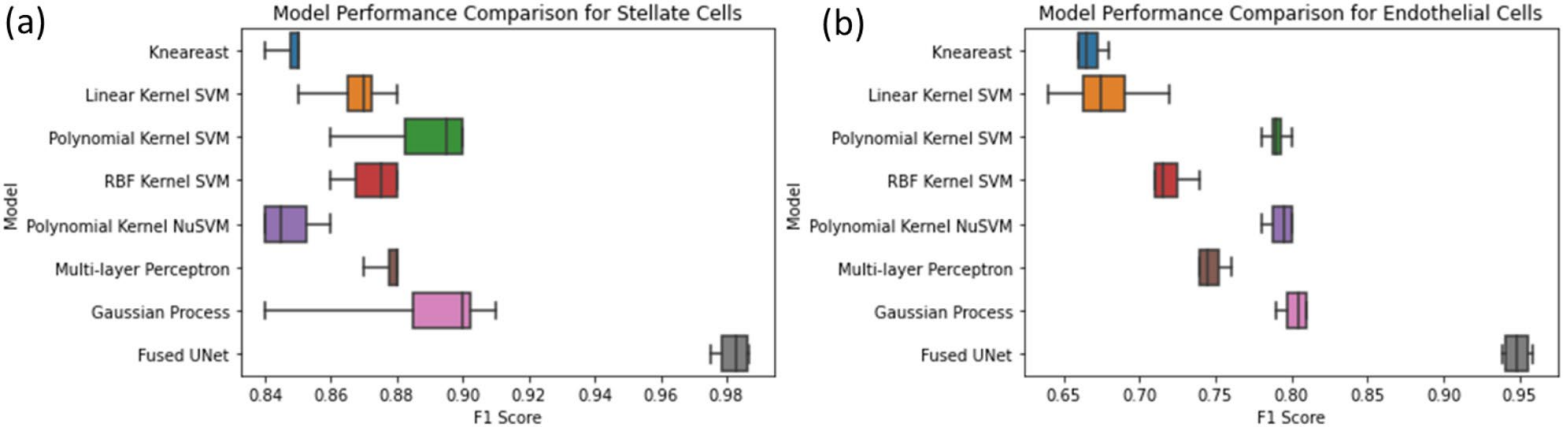
(**a**) Model performance comparison for HSC cells characterization; (**b**) model performance comparison for LEC cells characterization.

For comparisons between the Fused CNN UNet and conventional machine learning, we also conducted the cell characterization based on the morphological features of HSC and LEC cells datasets using conventional machine learning techniques. Our experiment shows that conventional machine learning classification algorithms generally return relatively high F_1_ scores for the HSC cells dataset but yielded lower F_1_ scores for the LEC dataset. The best conventional classifier model based on morphological features is the Gaussian process classifier model. It returns a mean F_1_ score of 0.905 for the HSC cells dataset and a mean F_1_ score of 0.81 for the LEC cells dataset. Figure 7 shows the comparison between the Fused CNN UNets and different conventional machine learning methods. The Fused CNN UNet outperforms all conventional machine learning algorithms by a significant margin for detection of early-stage NASH.

## Discussion

The multimodal NASH prognosis using 3D/2D single-cell images of liver cells along with artificial intelligence is a unique approach that has not been pursued. We applied a customized convolutional neural network and showed excellent accuracy in identifying healthy vs. diseased subjects. Our approach adds a new dimension to NASH prognosis and can be used as a surrogate biomarker. Recently there has been an increase in research on identifying biomarkers of liver cells using circulating cells^31^ and gene expression using single-cell RNA sequencing. Combining these technologies with our approach will greatly enhance our comprehension of NASH.

Understanding NAFLD and NASH progression is critical because of its growing impact on public health. Morphological and textural changes likely represent a disease progression. For example, both HSC and LEC diseased subjects have high entropy indicating less differentiation and cell plasticity. If we increase our sample subject population to include F3/F4 stage NASH cells, this trend might reveal a trajectory in NASH prognosis and can be used to build efficient drug discovery models. We also noticed lower homogeneity and energy density in diseased HSC correlating to retinol loss^32,33^ and a slight increase in volume, area, and principal axis, indicating the cell proliferation in the activated state. We noticed a slight regression in the homogeneity and energy density plot of diseased LEC (EC74) correlating to the reduction in fenestrae due to capillarization and nucleus vacuolation^34–36^.

NASH is a very complicated disease due to multiple pathways, several fibrosis stages (F0–F4), the time it takes to progress, and comorbidities. The lack of good in-vivo and primary liver cell-based in-vitro (2D or 3D spheroid culture) models^37–40^, along with the lack of reliable biomarkers, makes it even more complicated to understand and replicate the pathophysiology and disease progression. Due to these limitations, there is an unmet clinical need to understand the molecular & structural level changes in LEC and HSC cells in NASH subjects. Our approach offers a promising opportunity to develop better predictive NASH models. There are several clinical implications of the findings reported in our study. First, assessing the state change from quiescent HSC to activated HSC at the early stage could help distinguish simple steatosis from early-stage NASH and possibly revert the fibrosis process. Second, better predictive models by utilizing morphometric and textural features at cellular level^41^ can be surrogate biomarkers to the researchers working on pre-clinical models. Third, we can also correlate the changes at the cellular level to the gene expression obtained from single-cell RNA sequencing and use them to develop high throughput drug efficacy screening platform. Optimization of therapeutic outcomes for NASH may be best realized by targeting single-cell level biomarkers rather than looking at the entire liver tissue.

The ability of ML to detect and stage NASH^42^ depends on the quality of features within the model and the data used for training. The best set of features may also depend on the population under study as the incidence of NASH can vary across different stages. Further studies focusing on a larger donor dataset, with subjects from different stages of NASH, and the effects of administrating drugs that influence the restoration and capillarization of the LEC and HEC phenotype, could reveal more insight. Also, our machine learning model could be improved by training on a larger dataset that includes a broader pathophysiological process associated with the progression of NASH. In addition, future analysis will be focused on the other critical cells Kupffer and hepatocytes from the liver to understand the alterations these cells experience during disease progression.

## Conclusion

Hepatic stellate cells (HSCs) and Liver endothelial cells (LECs) have demonstrated clinically relevant therapeutic alterations for the early identification of NASH. These alterations were detected by incorporating label-free image-based biomarkers and customized UNet CNN for cell characterization. The proposed method shows the potential to segment and classify healthy vs. diseased NASH with high accuracy and robustness. Machine learning algorithm performance was validated using an independent test dataset of HSC and LEC cells. Results show 98% accuracy for overall cell detection. Finally, utilizing transmission and side scattered images generated by a 3D imaging flow cytometer (3D-IFC) and the morphometric and texture characteristics extracted from these images could produce surrogate label-free biomarkers to resolve different stages of NASH progression.

## Supplementary Information

Supplementary Information.

## Data Availability

Matlab scripts enabling the main steps of the analysis are available from the authors upon request. The Pytorch implementation of all deep learning models and training code is publicly available on Github: https://github.com/rut011/LiverCellML.
