# Supplementary material for: Multimodal NASH prognosis using 3D imaging flow cytometry and artificial intelligence to characterize liver cells
Source: Sci Rep. 2022 Jul 1;12:11180. doi: 10.1038/s41598-022-15364-7 (PMC9249889; doi:10.1038/s41598-022-15364-7)

**Multimodal NASH prognosis using 3D imaging flow cytometry and artificial intelligence to characterize liver cells**

# Supplementary Figures


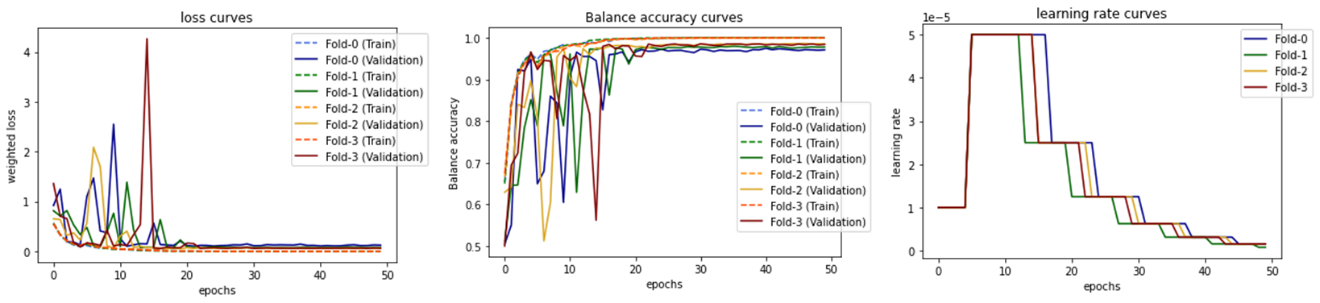


Supplementary Figure 1. Fused UNet Learning curves and learning rate schedules during training for stellate cells characterization


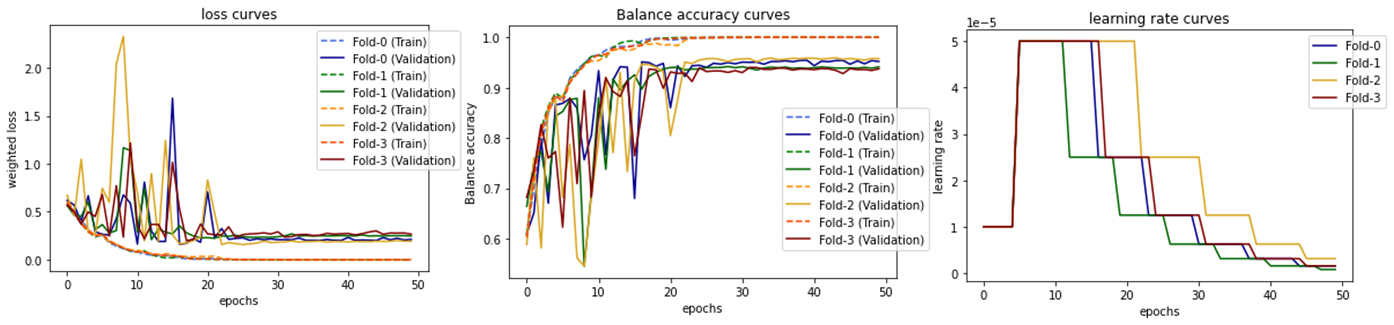


Supplementary Figure 2. Fused UNet Learning curves and learning rate schedules during training for endothelial cells characterization


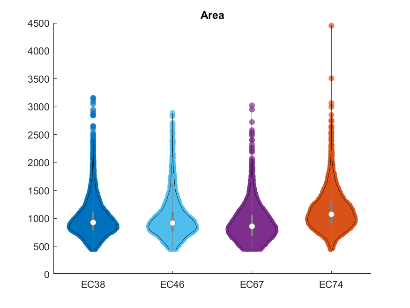

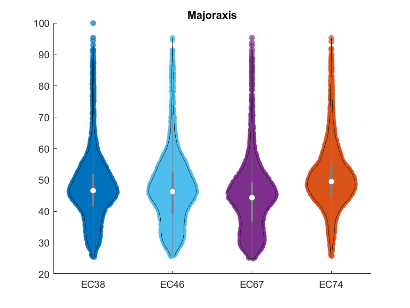

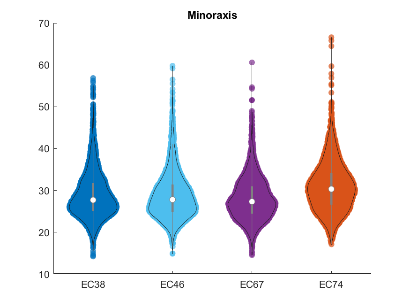

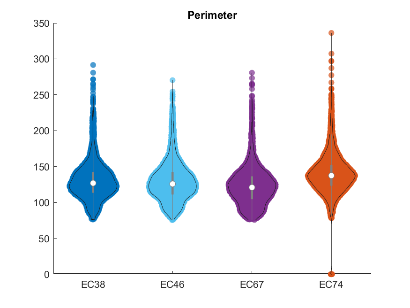

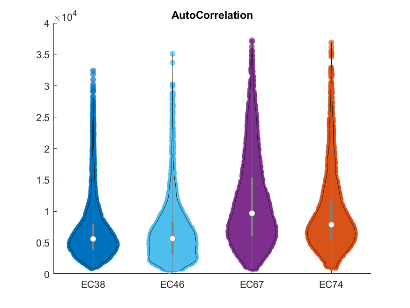

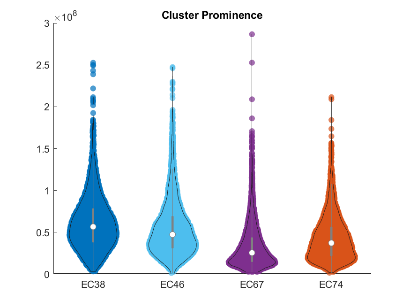

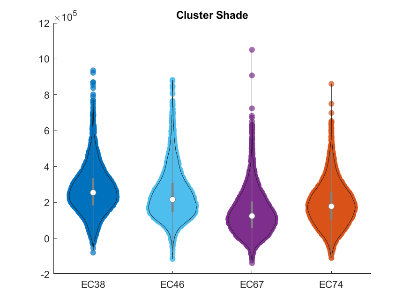

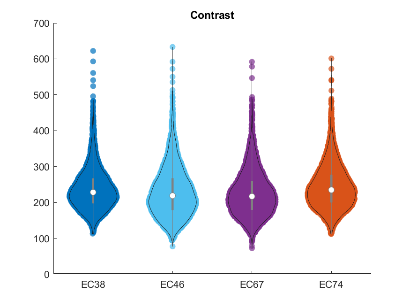

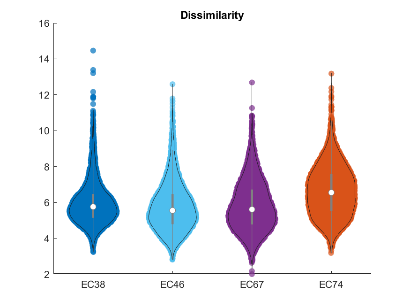


Supplementary Figure 3. Harlick texture and geometrical features comparison of Endothelial healthy vs diseased cells using 2D transmission image.


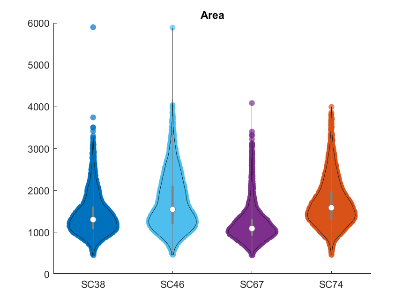

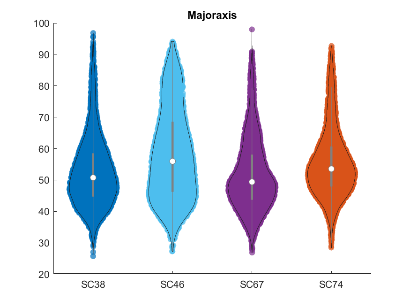

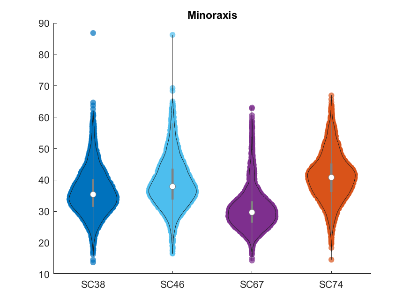

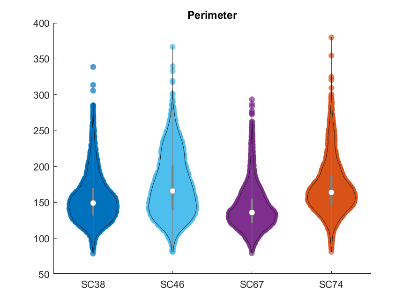

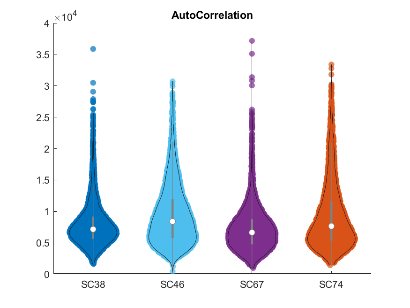

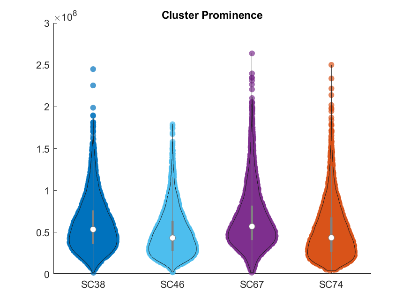

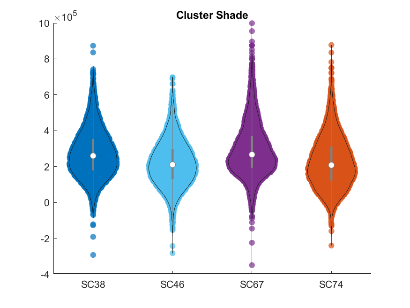

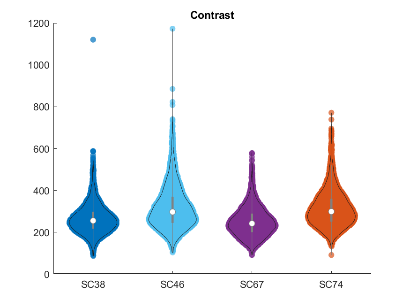

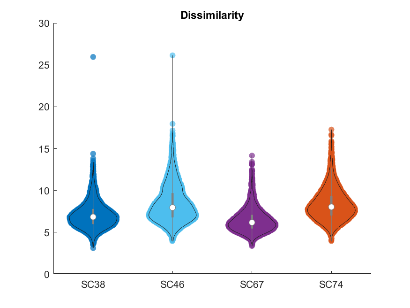


Supplementary Figure 4. Harlick texture and geometrical features comparison of Stellate healthy vs diseased cells using 2D transmission image.


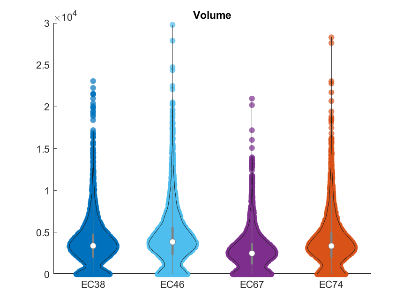

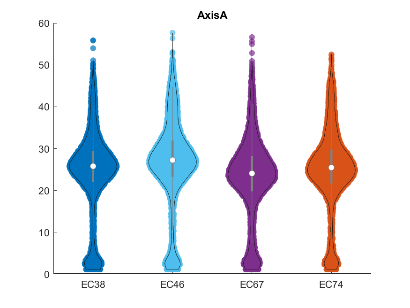

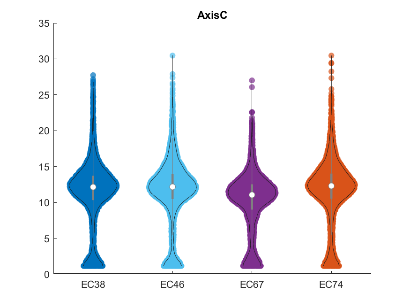

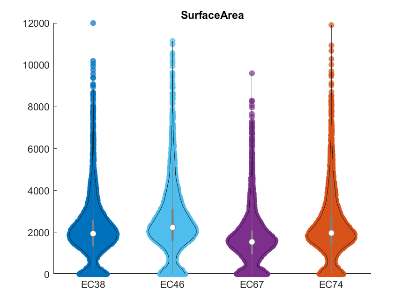

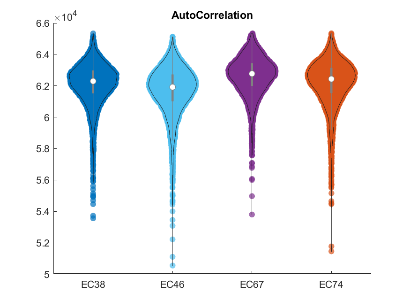

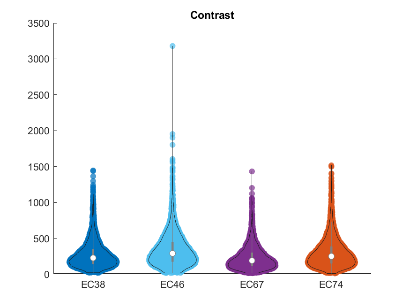

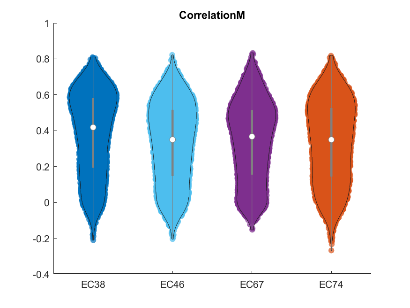

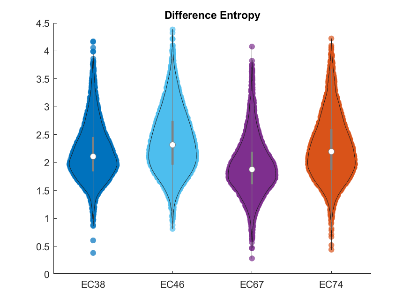

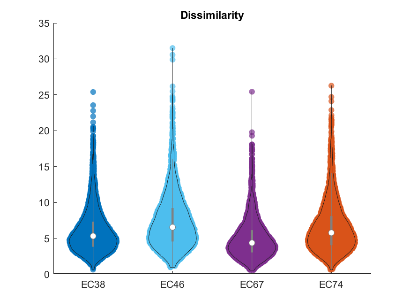


Supplementary Figure 5. Harlick texture and geometrical features comparison of Endothelial healthy vs diseased cells using 3D side scatter image.


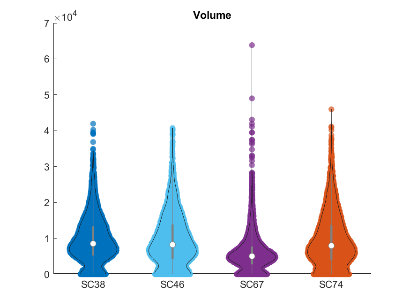

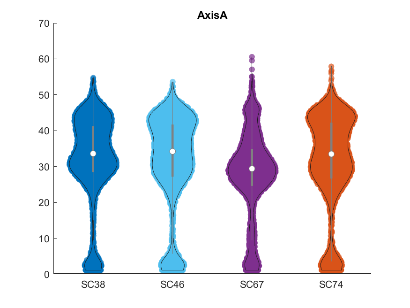

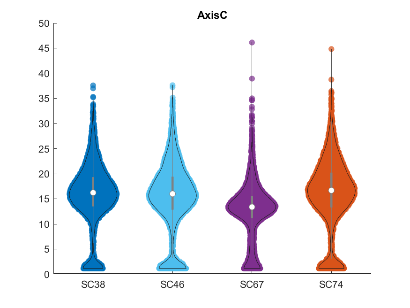

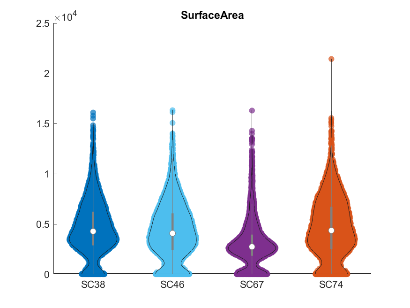

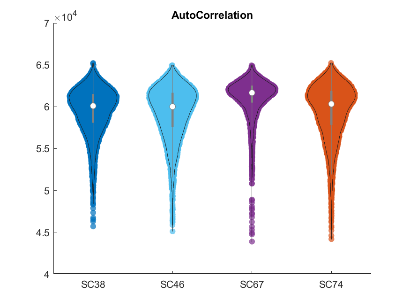

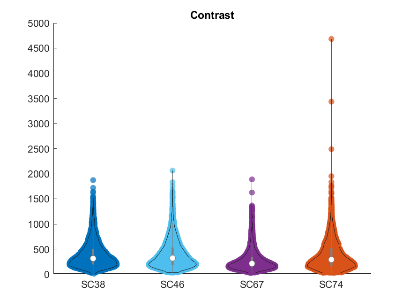

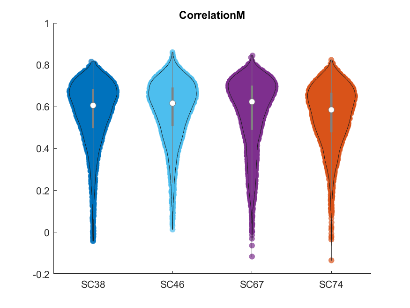

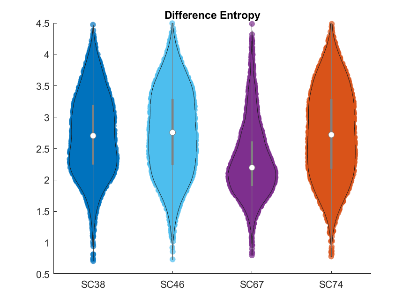

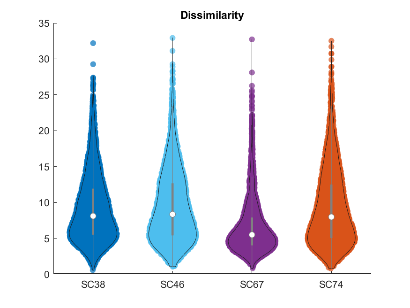


Supplementary Figure 6. Harlick texture and geometrical features comparison of Stellate healthy vs diseased cells using 3D side scatter image.

# Supplementary Tables

Supplementary Table 1. List of morphological features extracted from images acquired by the 3D-IFC system

| 2D transmission features | Autocorrelation, Contrast, Correlation, Cluster prominence, Cluster Shade, Dissimilarity, Energy, Entropy, Homogeneity, Maximum probability, Area, Major Axis, Minor Axis, Axis ratio, Circularity, Perimeter. |
| --- | --- |
|  |  |
| 3D side-scattering features | Autocorrelation, Contrast, Correlation, Cluster prominence, Cluster Shade, Dissimilarity, Energy, Entropy, Homogeneity, Maximum probability, Volume, Axis a, Axis b, Axis C, Surface Area, Volume/Surface Area. |
|  |  |

Supplementary Table 2. Training results summary of conventional machine learning classifier models for stellate cells dataset


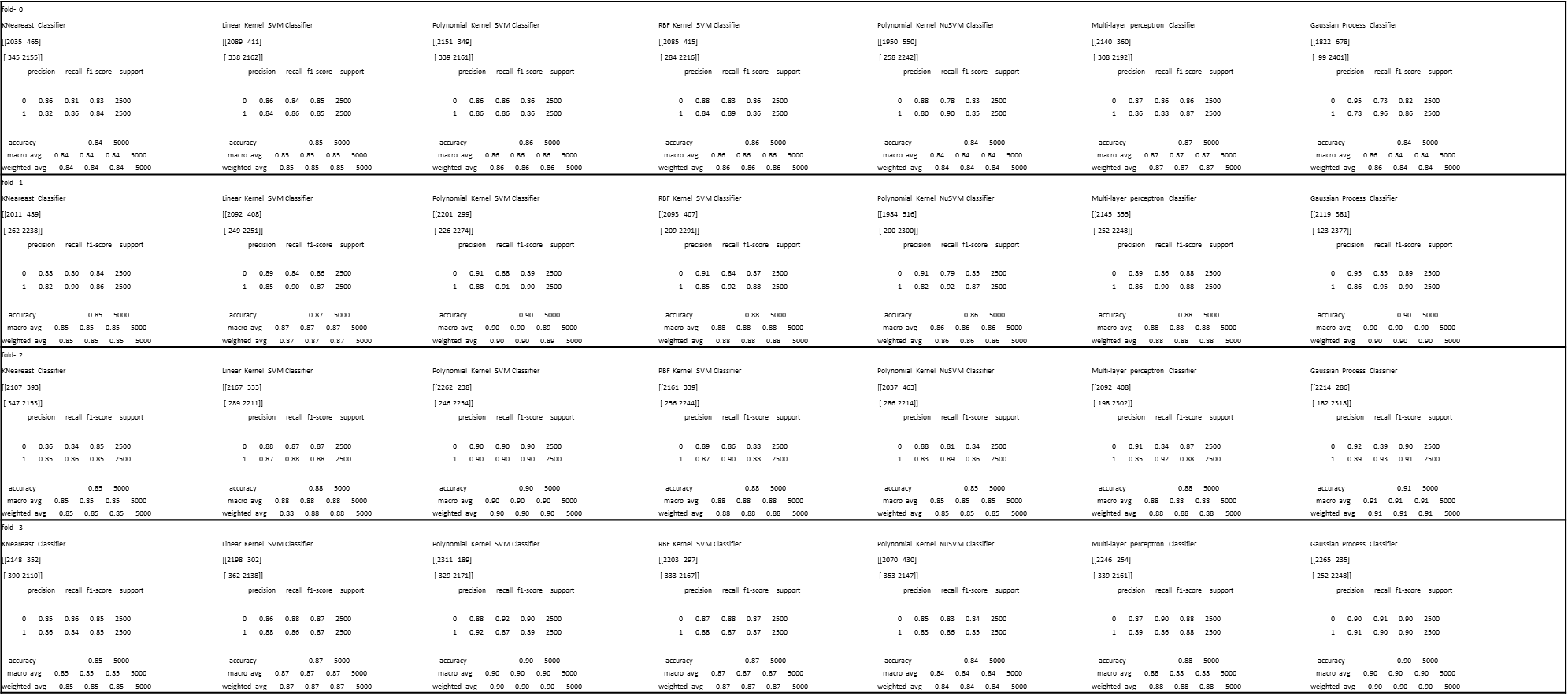


Supplementary Table 3. Training results summary of conventional machine learning classifier models for endothelial cells dataset


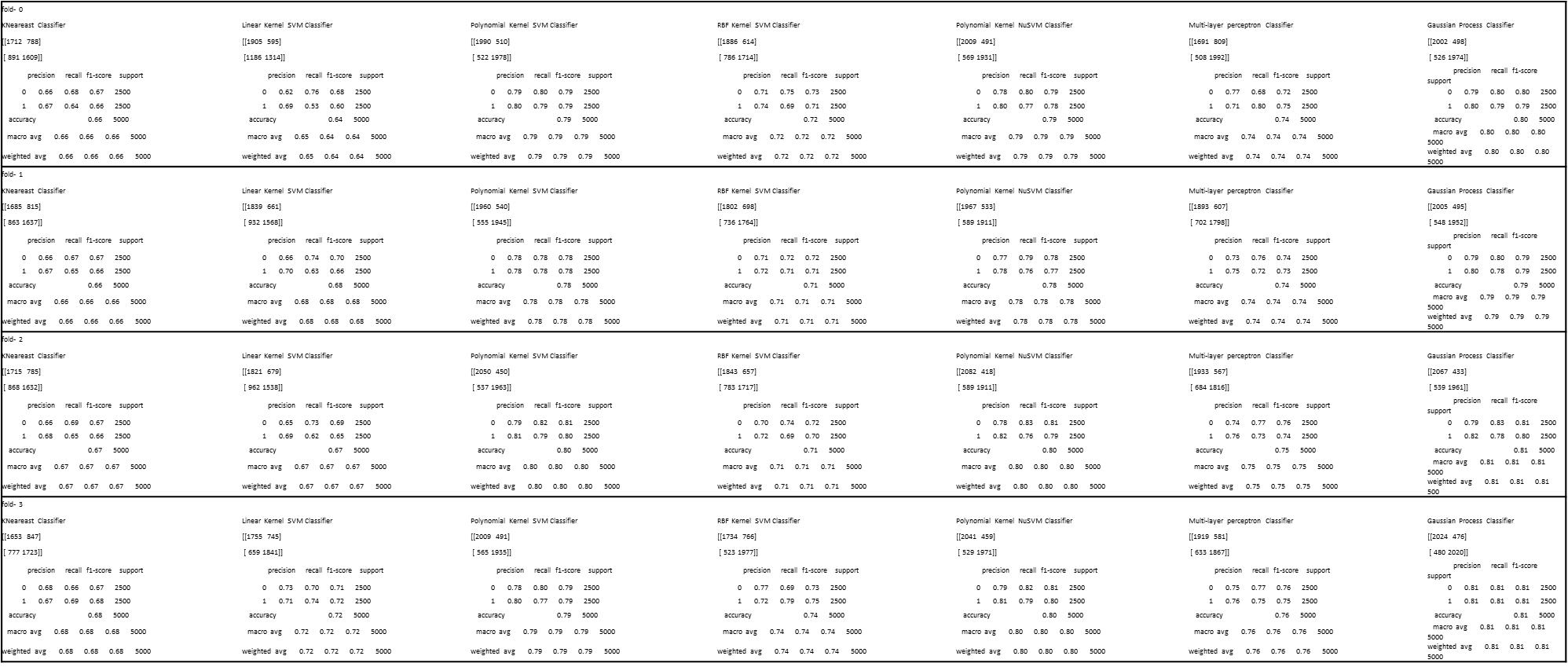

Supplement: Supplementary file 1 — Supplementary Information. [file 41598_2022_15364_MOESM1_ESM.docx]
